# The clock gene *Bmal1* inhibits macrophage motility, phagocytosis, and impairs defense against pneumonia

**DOI:** 10.1073/pnas.1915932117

**Published:** 2020-01-03

**Authors:** Gareth B. Kitchen, Peter S. Cunningham, Toryn M. Poolman, Mudassar Iqbal, Robert Maidstone, Matthew Baxter, James Bagnall, Nicola Begley, Ben Saer, Tracy Hussell, Laura C. Matthews, David H. Dockrell, Hannah J. Durrington, Julie E. Gibbs, John F. Blaikley, Andrew S. Loudon, David W. Ray

**Affiliations:** ^a^Faculty of Biology, Medicine, and Health, Manchester Academic Health Sciences Centre, University of Manchester, M13 9PT Manchester, United Kingdom;; ^b^Manchester Foundation Trust, Manchester Academic Health Science Centre, M13 9WL Manchester, United Kingdom;; ^c^National Institute for Health Research, John Radcliffe Hospital, Oxford Biomedical Research Centre, OX3 9DU Oxford, United Kingdom;; ^d^Oxford Centre for Diabetes, Endocrinology and Metabolism, University of Oxford, OX37LE Oxford, United Kingdom;; ^e^Leeds Institute of Cancer and Pathology, Faculty of Medicine and Health, University of Leeds, LS9 7TF Leeds, United Kingdom;; ^f^Department of Infection Medicine and Medical Research Council Centre for Inflammation Research, University of Edinburgh, EH16 4TJ Edinburgh, United Kingdom

**Keywords:** circadian, *Streptococcus pneumoniae*, phagocytosis, actin cytoskeleton, RhoA

## Abstract

The circadian clock regulates many aspects of immunity. Bacterial infections are affected by time of day, but the mechanisms involved remain undefined. Here we show that loss of the core clock protein BMAL1 in macrophages confers protection against pneumococcal pneumonia. Infected mice show both reduced weight loss and lower bacterial burden in circulating blood. In vivo studies of macrophage phagocytosis reveal increased bacterial ingestion following *Bmal1* deletion, which was also seen in vitro. BMAL1^−/−^ macrophages exhibited marked differences in actin cytoskeletal organization, a phosphoproteome enriched for cytoskeletal changes, with reduced phosphocofilin and increased active RhoA. Further analysis of the BMAL1^−/−^ macrophages identified altered cell morphology and increased motility. Mechanistically, BMAL1 regulated a network of cell movement genes, 148 of which were within 100 kb of high-confidence BMAL1 binding sites. Links to RhoA function were identified, with 29 genes impacting RhoA expression or activation. RhoA inhibition restored the phagocytic phenotype to that seen in control macrophages. In summary, we identify a surprising gain of antibacterial function due to loss of BMAL1 in macrophages, associated with a RhoA-dependent cytoskeletal change, an increase in cell motility, and gain of phagocytic function.

## Significance

The circadian clock extensively regulates physiology, with an emerging role in immunity. Bacterial infection development, progression, and resolution depend on the time of day, but through an unknown mechanism. Here, we show that time of day regulates macrophage phagocytosis, and that the core clock protein BMAL1 is responsible. BMAL1 regulates RhoA-dependent macrophage motility and bacterial engulfment, and loss of BMAL1 enhances antibacterial immunity. We identify a genetic circuit linking BMAL1 binding to motility, cytoskeletal gene expression, and RhoA activation. With the rise in antimicrobial resistance, finding new ways to enhance immunity, by targeting clock components, offers new therapeutic opportunities.

The regular 24-h environmental cycle generated by the planet’s rotation has led to the evolution of circadian rhythms in virtually all life forms on Earth. These are driven by autonomous cellular biological clocks, which coordinate physiology and behavior over the day–night cycle. In mammals, many physiological systems are regulated in a time-of-day–dependent manner. Included in this, mammalian immunity is strongly regulated by the circadian clockwork, driving the magnitude and nature of both innate and acquired responses (1). Macrophages, in particular, have a strong endogenous circadian clock, which drives inflammatory function (2–4). We have previously identified a role for the circadian clock in regulation of time-of-day variation in outcomes following pneumococcal infection (5), but the mechanisms responsible remain unknown.

The core cellular circadian pacemaker in mammals, oscillates with a 24-h period, and consists of a positive arm, comprising BMAL1/CLOCK heterodimeric transcription factors, which drive transcription of the repressor genes *period* and *cryptochrome*. As PERIOD and CRYPTOCHROME proteins accumulate, they then inhibit BMAL1/CLOCK transactivation function. In addition, BMAL1/CLOCK drive expression of a second repressor circuit consisting of the orphan nuclear receptors REVERBα, and REVERBβ. These, in turn, repress *Bmal1* gene expression. Beyond the core circadian transcription–translation feedback loop the core clock transcription factors act through clock-controlled genes to regulate many aspects of physiology, including energy metabolism and immunity (6).

Many cells of the innate immune system have intrinsic clocks, including monocytes, macrophages, neutrophils, mast cells, eosinophils, and natural killer cells (2, 7–13). These cell-autonomous rhythms drive aspects of differentiated cell function, including cytokine production, trafficking, and phagocytosis. Within myeloid cells the core circadian gene *Bmal1* exerts a broad antiinflammatory effect, mediated to a large extent through its transcriptional regulation of *Reverbα*, and *Reverbß* (3, 14, 15). Additional direct *Bmal1* effects in macrophages have also been identified (16, 17). For instance, specific bacterial infections show a time-of-day dependence in outcome, including enteric *Salmonella typhimurium* (18) and *Streptococcus pneumoniae* (5).

Infectious diseases are responsible for many deaths both in the developed and developing world and, in the case of pneumonia, are responsible for 5% of all deaths in the United Kingdom (19). The emergence of multidrug-resistant bacteria makes it essential that we gain a better understanding of the mechanisms behind infection, to identify new therapeutic strategies. The role of circadian biology in bacterial pathogenesis has yet to be fully explored. This is important, as several compounds now exist which can alter key circadian pathways as well as repress or accentuate circadian amplitudes. One of the key pathways governing the pathogenicity of an organism is phagocytosis.

Phagocytosis is the process of ingestion of large particles by cells, based on rearrangement of the actin microfilament cytoskeleton. Macrophages and neutrophils are typical cells that fulfill this function in mammals and are beneficial for host defense against bacterial pathogens. Engulfment of the particle, through cell-surface receptors for immunoglobulins, or complement, activates small GTP binding proteins of the Rho family, with specificity of downstream coupling. As an example RhoA is activated by the complement receptor, but not the FcγR receptor. In its active, GTP bound state, RhoA interacts with further downstream effectors to drive F-actin reorganization. Previous work suggests that ex vivo synchronized macrophages show time-of-day–dependent changes in *S. typhimurium* phagocytosis and bacterial killing (20). However, the role of the circadian clock and its components in regulating phagocytosis, and the impact this has on bacterial responses in vivo remains undefined. Here we identify a significant gain in pneumococcal immunity resulting from loss of the core clock protein BMAL1 in macrophages. This was accompanied by an increase in macrophage movement, and phagocytosis, but not by a change in immune cell infiltration to the infected lung. The most striking change was protection from bacteraemia (extension of the infection to the bloodstream). Further analysis identified a gain in RhoA, and cofilin activity, accompanied by a major reorganization in the actin cytoskeleton. This macrophage gain-of-function phenotype was reversed by low concentrations of the specific RhoA inhibitor CT04.

## Results

### *Bmal1* Deletion Increases Resistance to Pneumococcal Pneumonia.

**Fig. 1.**
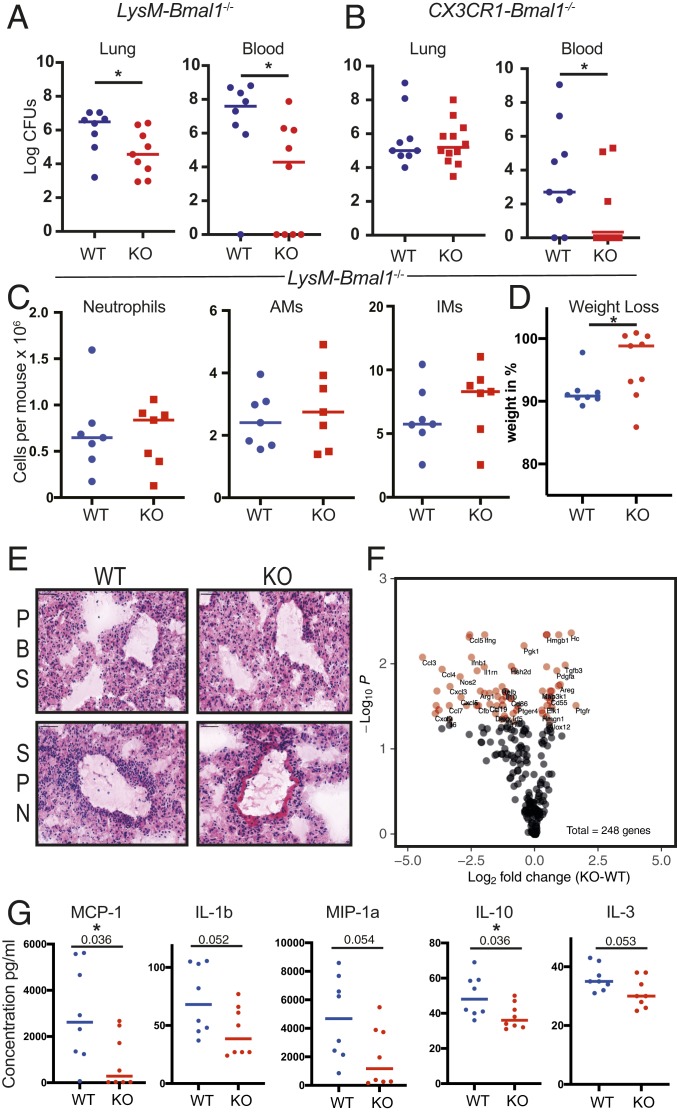
Deletion of BMAL1 in macrophages protects against pneumococcal infection. (*A*) *LysM-Bmal1*^*−/−*^ and (*B*) *CX3CR1-Bmal1*^−/−^ mice (knockout [KO]) and floxed littermate controls (WT) were infected with *S. pneumoniae* at ZT12. The bacterial load in lung and peripheral blood at 48 was quantified (*n* = 8 to 12, Mann–Whitney *U* test *P < 0.05). (*C*) Immune cells were recovered from the lung tissue in *A* neutrophils, alveolar macrophages (AMs), and interstitial macrophages (IMs). (*D*) Weight loss after 48 h (Mann–Whitney *U* test *P < 0.05). (*E*) Representative lung histology with H&E staining. (Scale bar, 50 µm.) SPN, *Streptococcus pneumoniae*. (*F*) Cytokine analysis of lung tissue from *A* was performed using a NanoString inflammatory gene panel (see also *SI Appendix*, Fig. S1*F*). Each of the 248 genes is marked, and those that statistically differ by genotype are marked in red, with gene abbreviation. (*G*) Multiplex cytokine analysis of tissue from *A* (*n* = 8, *t* test, exact *P* value marked, and *P ≤ 0.05).

Previous work has identified circadian control of the lung inflammatory response to an acute challenge with aerosolized lipopolysaccharide (5). Furthermore, this study demonstrated variation in the outcome from pneumococcal pneumonia (the most common form of pneumonia) dependent on time of inoculation. Time of day affects ex vivo phagocytic function of macrophages (21, 22), suggesting a macrophage-autonomous circadian control of phagocytosis. Therefore, to explore the role of the circadian clock within the myeloid system in lung immunity against infection, we utilized mice lacking the core clock component *Bmal1* in myelomonocytic cells (*LysM*-*Bmal1*^−/−^ alveolar macrophage penetrance confirmed *SI Appendix*, Fig. S1 *A*, *Left*) or macrophages (*CX3CR1*-*Bmal1*^−/−^, *SI Appendix*, Fig. S1 *A*, *Right*) (Fig. 1 *A* and *B*). Both mouse lines showed striking protection from bacteraemia (blood culture positive), 48 h after pneumococcal infection, but the *LysM-Bmal1*^*−/−*^ mice also showed reduced lung bacterial burden, and a trend toward reduced bacteraemia was seen at 24 h following inoculation (*SI Appendix*, Fig. S1*B*). There was no difference in total pulmonary immune cellularity (Fig. 1*C* and *SI Appendix*, Fig. S1 *C* and *D*), nor significant differences in peripheral blood proinflammatory cytokine concentrations at 12 and 48 h postinfection (*SI Appendix*, Fig. S1*E*). Taken together these findings suggest that the antibacterial efficacy of each immune cell is greater, thereby preventing extension of the infection to the bloodstream with similar numbers of effector immune cells in the lung.

To test the idea that BMAL1 loss in macrophages provides systemic protection from pneumonia, we also measured weight loss, a measure of the systemwide impact of sepsis (Fig. 1*D*). Mice lacking macrophage BMAL1 showed markedly less weight loss than the littermate controls. Histological examination of the affected lungs showed a similar immune cell infiltrate in both genotypes, in accordance with the flow cytometry enumeration of immune cells (Fig. 1*E*). NanoString measurement of inflammatory mediator gene expression showed reduced proinflammatory cytokines and increased expression of growth factors in animals lacking macrophage BMAL1, indicating a proresolution state compared to the littermate controls (Fig. 1*F* and *SI Appendix*, Fig. S1*F*). The proposed reduction in inflammatory mediator expression in the lungs was further tested using a bead array to measure protein concentrations in recovered lung tissue. There was a general trend for lower concentrations of both pro and antiinflammatory cytokines, with CCL2, the monocyte chemokine, and IL10, an important antiinflammatory cytokine, both showing a significant reduction (Fig. 1*G*).

We have previously identified the orphan nuclear receptor REVERBα as an obligate mediator of many of the inflammatory effects resulting from BMAL1 loss in the macrophage (3). Accordingly we tested myelomonocytic disruption of the Reverbα gene, using the reported DBD^m^ strain crossed with LysM-cre (23) (*SI Appendix*, Fig. S1*G*). Surprisingly, we saw no phenotype in terms of bacterial outcome in lung, or in blood. Further, we exposed wild-type (WT) animals to continuous light, which attenuates circadian oscillations (24, 25). This also did not impact outcomes to pneumococcal infection (*SI Appendix*, Fig. S1*H*).

### *Bmal1* Deletion Increases Phagocytic Function in Macrophages.

**Fig. 2.**
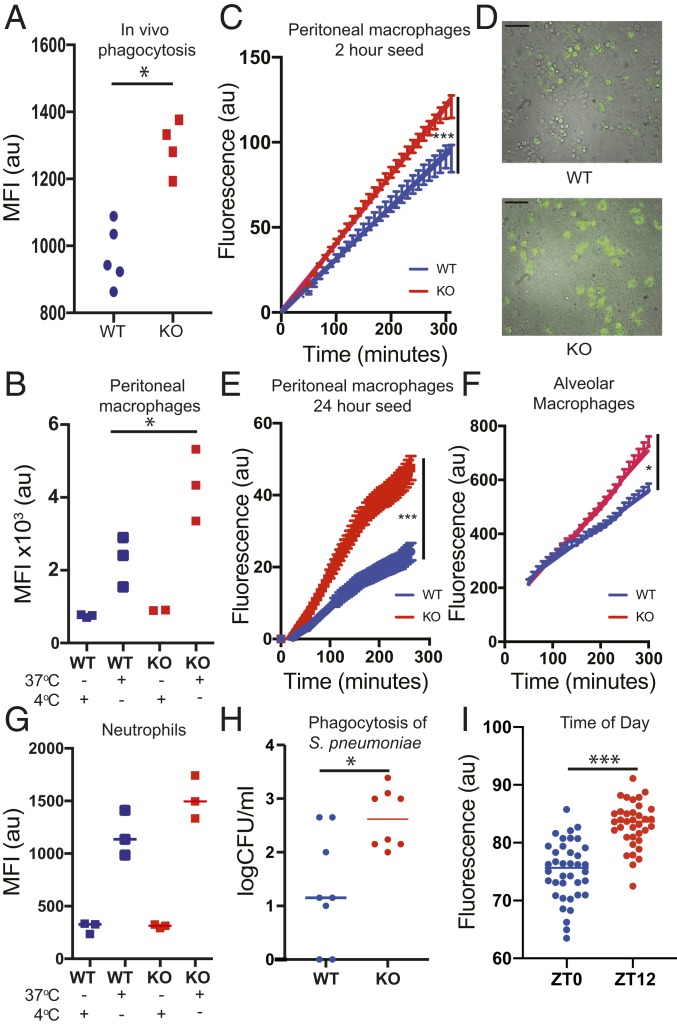
BMAL1 deletion improves macrophage phagocytosis. (*A*) *LysM-Bmal1*^*−/−*^ mice and floxed controls were infected i.p. with *S. aureus* pHrodo green (SAPG). Peritoneal exudate cells were sampled after 45 min and subjected to FACS. The MFI was quantified (WT *n* = 5, *LysM-Bmal1*^*−/−*^ *n* = 4). *P < 0.05; Mann–Whitney *U* test. (*B*) Ex vivo phagocytosis assay. Peritoneal macrophages were incubated with SAPG for 4 h, before FACS analysis (gating strategy see *SI Appendix*, Fig. S1*D*). (*n* = 3 mice) (Mann–Whitney *U* test *P ≤ 0.05). Cells incubated with SAPG at 4 °C to inhibit enzymatic function in the macrophages are also shown. (*C*–*E*) Ex vivo live cell microscopy phagocytosis assays with representative combined brightfield and fluorescence image. Peritoneal macrophages were recovered from *LysM-Bmal1*^*−/−*^ mice and floxed control mice, and seeded for (*C* and *D*) 2 h (WT, *n* = 48) positions, *LysM-Bmal1*^*−/−*^, *n* = 57 positions, quantification and representative fluorescent microscopy are shown. (Scale bar, 25 µm.) (*E*) At 24 h (WT *n* = 25 positions, *LysM-Bmal1*^*−/−*^ *n* = 30 positions). SAPG was added to the cultures and the increase in MFI measured (mean and SEM plotted repeated measures ANOVA ***P < 0.0001). For representative FACS plot see *SI Appendix*, Fig. S2*D*. (*F*) Alveolar macrophage live cell microscopy phagocytosis assay. (*n* = 10 positions). (*G*) Ex vivo neutrophil phagocytosis assay. Bone marrow-derived neutrophils from *LysM-Bmal1*^*−/−*^ were plated in vitro and subject to the same phagocytosis protocol as *B*. After 4 h, there was no genotype effect (*P* = 0.2, Mann–Whitney *U* test). (*H*) Ex vivo *S. pneumoniae* phagocytosis assay. Peritoneal macrophages were incubated with *S. pneumoniae*, D39. At 4 h after challenge, viable intracellular bacteria were assessed (*n* = 8, Mann–Whitney *U* test *P ≤ 0.05). (*I*) Peritoneal macrophages from C57BL/6 mice were subjected to live cell phagocytosis assay with labeled *S. aureus* at ZT0 and ZT12. (*n* = 36 to 38 wells per time of day) (Mann–Whitney *U* test ***P < 0.0001).

To test the intrinsic macrophage antibacterial activity, isolated macrophages were incubated with a readily ingested bacterial pathogen, pHrodo-transgenic *Staphylococcus aureus* (26). Due to the inherent properties of the pHrodo dye, ingested bacteria fluoresce when the phagosome fuses with the acidic lysosome compartment (27). There was a significant increase in formation of phagolysosomes, per macrophage, in response to *Bmal1* deletion, both when tested in vivo through intraperitoneal (i.p.) injection, or ex vivo, in culture (Fig. 2 *A* and *B*). Using live cell imaging the kinetics of the response are clear. We can see isolated macrophages responding to bacteria with phagocytosis and observed greater phagocytosis at all time points, but with a significant difference emerging even by 2 h postseeding. (Fig. 2 *C*–*E*) (28). To investigate a possible role for secreted factors in augmenting phagocytosis, we tested the impact of conditioned medium from BMAL1-null macrophages, but this revealed no effect, pointing to a cell-autonomous mechanism of BMAL1 action (*SI Appendix*, Fig. S2*A*). The impact of increased bacterial ingestion is readily seen in fluorescent imaging, where enlarged macrophages laden with fluorescent bacteria are seen in the absence of BMAL1 (Fig. 2*D*).

We returned to study alveolar macrophages purified from the lung and analyzed ex vivo using the optimized protocols described above (Fig. 2*F*). Again, the increased ingestion of bacteria was seen in the absence of BMAL1, indicating this to be a generalized macrophage phenotype. As some of our studies used the *LysM-*cre driver, which also targets neutrophils, we purified neutrophils from bone marrow and tested them in vitro, using the same pHrodo-labeled *S. aureus* technology (Fig. 2*G*). Although there was a trend for increased phagocytosis in the absence of BMAL1, this was not significant, and the magnitude of effect was small. Therefore, the major driver of altered bacterial response in BMAL1 deletion is the macrophage population. Because the in vitro analysis used labeled *S. aureus* rather than *S. pneumoniae*, we also tested phagocytosis of *S. pneumoniae* in isolated macrophages in vitro using a gentamicin protection assay, and as with the *S. aureus*, we found this was increased in the absence of BMAL1, indicating this is a more generalized antibacterial property of the modified macrophages (Fig. 2*H*) resulting in increased phagocytosis. As these studies had revealed such a striking impact of BMAL1 on macrophages, we tested the effect of circadian phase on the macrophage phagocytic phenotype, as previous reports had suggested differences dependent on circadian phase. Here, we did see that phagocytosis was significantly greater at zeitgeber time (ZT)12 compared to ZT0 (Fig. 2*I*).

We saw no impact of either disruption of the REVERBα DNA binding domain, nor complete REVERBα deletion on isolated macrophage phagocytosis (*SI Appendix*, Fig. S2*B*), supporting the lack of in vivo pneumococcal infection phenotype. Further analysis using the REVERB ligand GSK4112, which targets both REVERB paralogs, had no effect on phagocytosis (*SI Appendix*, Fig. S2*C*).

### BMAL1 Expression Drives a Distinct Macrophage Phenotype.

**Fig. 3.**
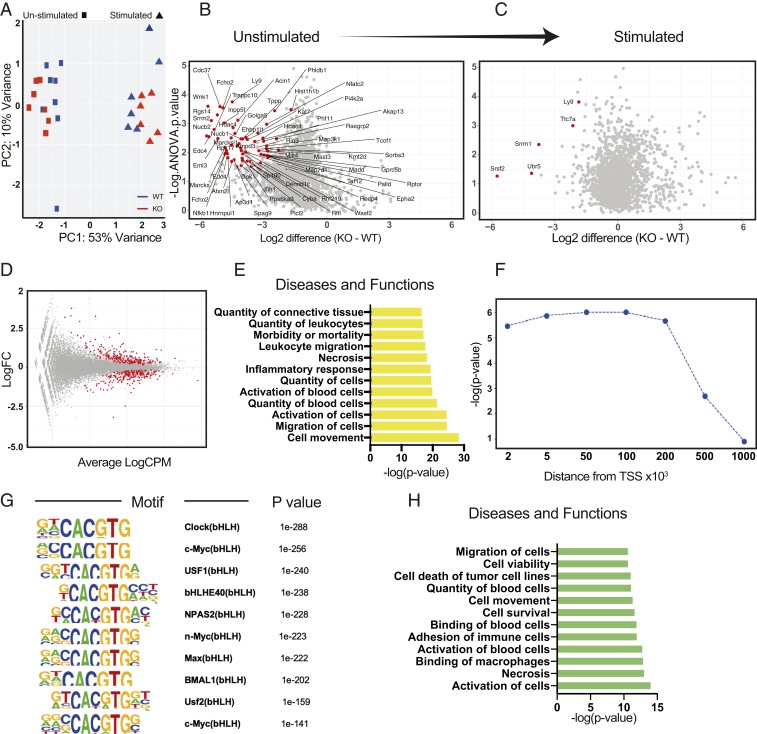
BMAL1 deletion in macrophages preactivates the cells. (*A*) Phagocytosis gene array in peritoneal macrophages using NanoString technology. Peritoneal macrophages were purified from *LysM-Bmal1*^*−/−*^ and floxed littermate controls mice, and cultured in vitro for 24 h, and then 100 µg/mL *S. aureus* or control media was added for 1 h. RNA was purified and analyzed by NanoString, using a phagocytosis panel (*SI Appendix*, Table S1). The principal component analysis (PCA) plot comparing stimulated and control cells from both of the two genotypes is shown, with the *LysM-Bmal1*^*−/−*^ (red) and floxed littermate controls (blue). Each point represents an individual mouse. (*B* and *C*) Peritoneal macrophages from *LysM-Bmal*^*−/−*^ and floxed littermate controls harvested at ZT2 were cultured for 24 h and then treated with 100 µg/mL *S. aureus* for 30 min (stimulated) or vehicle control (unstimulated). Cells were harvested and phosphopeptides purified. On the volcano plots, significant changes in identified phosphorylation sites are shown in red. Negative *x* axis values indicate lower in BMAL1^−/−^ cells (identified by ANOVA with post hoc test; *n* = 3). (*D*) RNA-Seq in unstimulated peritoneal macrophages. Magnitude-Average (MA) plot showing 454 genes with false discovery rate (FDR) < 0.01, in red. Cpm, counts per million. (*E*) Top IPA diseases and functions terms for the significant DE genes in *D*. (*F*) Enrichment of RNA-Seq DE genes in relation to BMAL1 binding sites for different genomic distances from the center of the BMAL1 binding peaks. (*G*) Top 10 motifs found using the BMAL1 peaks overlapping with DE genes at ±100-kb distance. (*H*) Top IPA diseases and functions terms for the 148 BMAL1 direct target genes.

In an unbiased gene expression analysis of 84 phagocytosis mediators, there was no difference seen under unstimulated conditions between control and BMAL1-null peritoneal macrophages, and both genotypes showed a similar major change in gene expression when activated (Fig. 3*A*). This suggests that the functional difference between the genotypes lies at the level of posttranslational modification of the phagocytosis machinery, rather than at the level of phagocytic gene expression. Phosphorylation is known to play a major role in circadian control of physiology (29). Therefore, we analyzed the phosphoproteomic changes between BMAL1-null and control macrophages, and measured these before and immediately after bacterial exposure. Here, we saw a striking difference between the genotypes under resting conditions, with a marked reduction in detected phosphosites in the BMAL1-null macrophages (Fig. 3*B*), but negligible differences remain after bacterial encounter (Fig. 3*C*). This is further illustrated by within-genotype analysis where the greatest difference following stimulation is seen in the control cells compared to the BMAL1-null macrophages, where macrophage activation prior to stimulation means fewer phosphosites change following bacterial stimulation (*SI Appendix*, Fig. S3*A*). Network analysis (*SI Appendix*, Fig. S3*B*) highlights the major pathways involved in cell motility, and phagocytosis, including the actin cytoskeleton emerged as differentially phosphorylated, and thereby activated, between the genotypes (*SI Appendix*, Fig. S3*C*).

In order to identify how BMAL1 affects cell motility and phagocytosis, we took an unbiased approach, purified peritoneal macrophages, and ran RNA-Seq analysis, comparing the two genotypes under resting conditions (Fig. 3*D*). The top gene ontology terms identified in differential gene expression analysis were cell movement and migration of cells (Fig. 3*E*). Although macrophage phagocytosis was delivered as a significant term it was far less significant, suggesting that the macrophage motility may be a dominant aspect of the phenotype rather than the mechanics of bacterial engulfment, explaining the failure of the original NanoString analysis to identify phagocytosis gene expression differences in the absence of BMAL1. Using Ingenuity pathway analysis (IPA) (Qiagen), we identified the differentially regulated genes within the cell movement pathway (Fig. 3 *D* and *E* and Dataset S2). Next, we performed an integrative analysis of the differentially expressed genes from the RNA-Seq and the binding sites identified in previously published peritoneal macrophage BMAL1 chromatin immunoprecipitation (ChIP)-Seq dataset (30). Here, we extend BMAL1 binding sites in both directions and extract the nearest coding genes. Taking these genes, whose transcription start sites (TSSs) overlap with BMAL1 binding sites for a given genomic distance, we calculate statistical enrichment of our differentially regulated geneset (those that change in the BMAL1-null macrophages). In this way we identify strong enrichment of our DE genes up to 100 kb from the BMAL1 binding sites after which the statistical significance declines (Fig. 3*F*). Therefore, we use the 100-kb distance cutoff either side of BMAL1 binding sites, which gives us a 148-gene subset from our DE geneset. Motif analysis of the corresponding BMAL1 peaks produces multiple E-box motifs as top hits, as would be predicted for BMAL1 binding sites (Fig. 3*G*). IPA analysis of these 148 genes found by using this integrative analysis in terms of diseases and functions yields strong motility and phagocytosis-related terms (Fig. 3*H* and Dataset S3).

### *Bmal1* Deletion Alters Actin Cytoskeletal Structures and Increases Movement in Macrophages.

**Fig. 4.**
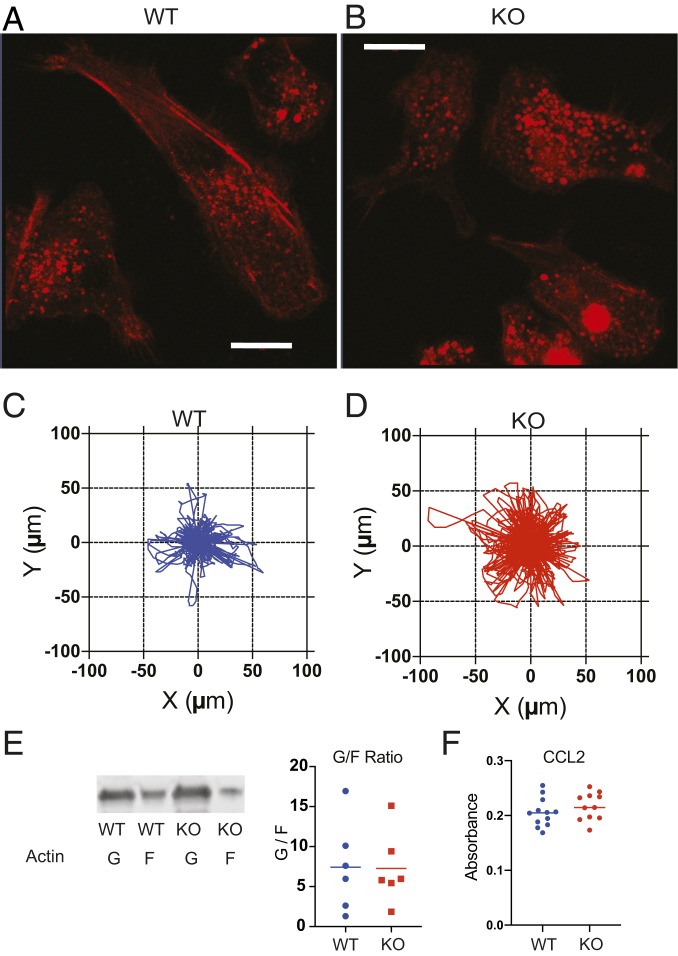
BMAL1 deletion alters macrophage morphology and motility. (*A* and *B*) Peritoneal macrophages from *LysM-Bmal1*^*−/−*^ and floxed littermate controls. Live cell imaging with ultraresolution confocal microscopy, using Airyscan detector showing F-actin. (Scale bar, 10 µm.) (*C* and *D*) Macrophages were also tracked through spontaneous migration for 4 h using a Livecyte kinetic cytometer (phasefocus). The distance moved and direction are represented in the rose plots. Mean step length WT 3.81 µm, and KO 5.04 µm; median step length WT 2.24 µm, and KO 3.61 µm; unpaired *t* test, *P* value < 0.0001 (cell number WT *n* = 683, KO *n* = 870 combination of cells from 12 wells per genotype). (*E*) Macrophages (WT and KO) were cultured for 72 h postrecovery from animals before lysis and analysis of globular and filamentous actin. The different molecular forms were quantified by immunoblot and plotted. There was no genotype effect. (*F*) Transwell migration assay. Peritoneal macrophages were cultured in vitro at 1 × 10^5^ cells per 24-well plate in a Transwell insert. Migration across the membrane toward CCL2 at 500 ng/µL was quantified using crystal violet staining after 4 h (Mann–Whitney *U* test; NS *P* > 0.05).

As the actin cytoskeleton emerged as a strong target for BMAL1 in macrophages, cell morphology was examined more closely. There was a dramatic difference in appearance, with loss of cortical actin, and marked cell rounding seen in the BMAL1-null macrophages (Fig. 4 *A* and *B* and Movies S1 and S2). This implies a more motile phenotype, and indeed the BMAL1^−/−^ cells moved a significantly greater distance when tracked in real time (Fig. 4 *C* and *D* and *SI Appendix*, Fig. S4 *A*–*D*), without directionality. However, we did not see a steady-state difference in G/F-actin ratio between the genotypes (Fig. 4*E*). Macrophages exhibit coordinated migration along chemokine gradients. In order to determine if the increased cell motility was translated into greater velocity along a CCL2 chemokine gradient, we used a Boyden chamber chemotaxis assay (Fig. 4*F* and *SI Appendix*, Fig. S4*E*). This did not show any genotype difference, identifying a rather specific change in macrophage phenotype that was confined to motility, and which did not impact migratory response to chemokine signal.

**Fig. 5.**
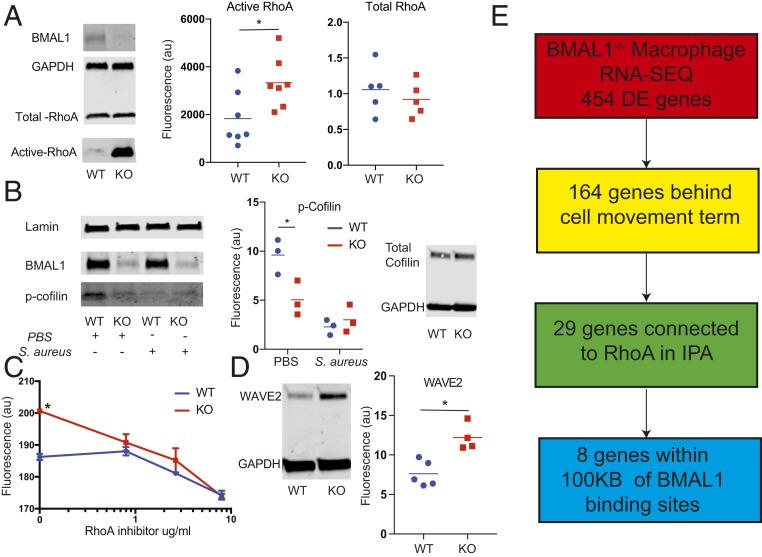
*Bmal1* deletion increases RhoA activation, and inhibition of RhoA rescues the phagocytosis phenotype. (A) Peritoneal macrophages from *LysM-Bmal1*^*−/−*^ (KO) and floxed littermate controls (WT) were cultured under control conditions for 24 h after harvest. Cells were lysed and analyzed for BMAL1, GAPDH, total RhoA, and active RhoA. Active and total RhoA abundance was quantified, after normalization to GAPDH. *P < 0.05. (*B*) Macrophages (WT and KO) were activated with heat-killed *S. aureus* for 30 min before protein lysate was analyzed for phospho, and total cofilin, with BMAL1, and lamin as controls; *P < 0.05 by Mann–Whitney *U* test. (*C*) Macrophages (WT and KO) were cultured for 24 h postrecovery from animals and then subjected to a live cell phagocytosis assay with labeled *S. aureus*. Cells were incubated with the CT004 RhoA inhibitor at the indicated concentrations from 1 h before adding bacteria (*n* = 4 per dose). Mean and SD are shown; *P < 0.05 by Mann–Whitney *U* test. (*D*) Macrophages (WT and KO) were cultured for 24 h postrecovery from animals under control conditions before lysis and analysis for WAVE2 expression by immunoblot; *P < 0.05 by Mann–Whitney *U* test. (*E*) Schematic diagram showing how 164 genes involved in the cell movement term (Fig. 3*E* and Dataset S2) connect to RhoA in IPA and those with transcription start sites within 100 kb of BMAL1 peaks.

The RhoA signaling pathway is a major regulator of the actin cytoskeleton and is activated in macrophages by complement-opsonized bacteria via complement receptor 3. Therefore we performed phagocytosis experiments with serum and analyzed RhoA activity. Indeed, loss of BMAL1 markedly increased RhoA activation under resting conditions (Fig. 5*A*). To investigate the remodelling of the actin cytoskeleton further, we analyzed phosphocofilin, a major regulator of actin depolymerization, and identified that the bacterial activation of macrophages decreased phosphocofilin in control cells, but that in the BMAL1-null macrophages phosphocofilin was already low (Fig. 5*B*). Overall our data suggest that BMAL1 acts to maintain macrophages in a quiescent state. When BMAL1 is removed, we see a change in macrophage phenotype, with RhoA activation, a remodelled cytoskeleton, increased cell motility, and increased phagocytosis. To test the idea that basal induction in RhoA is sufficient to prime BMAL1-null macrophages for enhanced bacterial phagocytosis, we used a RhoA inhibitor (CT04) (31). In this study the inhibitor was titrated into a phagocytosis assay, and the impact measured. Again, we observed the basal increase in phagocytosis in the BMAL1-null macrophages, but now see that this is reduced to the level of wild-type activity in the BMAL1-null macrophages in response to low-dose RhoA inhibition, at a concentration insufficient to affect wild-type cells (Fig. 5*C*). At higher concentrations both genotypes of macrophages show a reduction in bacterial ingestion, with no genotype differences.

We investigated the downstream mediators of activated small GTPases in the targeted macrophages and, informed by the analysis of phosphoproteins (*SI Appendix*, Fig. S3*C*), saw a marked increase in basal expression of WAVE2 (Fig. 5*D*), an essential link between the GTPases and Arp2/3 regulation of actin branching, and polymerization, providing further evidence of a remodelled actin control machinery. Having identified a strong signal for differential activity in pathways affecting the actin cytoskeleton and Rho GTPases in the phosphoproteome analysis (*SI Appendix*, Fig. S3*C*), and discovering that cell movement was identified as the most significant “diseases and functions” term in the transcriptome (Fig. 3*E*) analysis, we next looked to identify regulatory links between BMAL1 and RhoA. The IPA term “cell movement” was delivered with 164 genes found to be differentially regulated. Of these genes, 29 genes have functional links to RhoA in IPA (*SI Appendix*, Fig. S5), with 8 of these genes also being located within 100 kb of high-confidence BMAL1 peaks (30) (Fig. 5*E* and *SI Appendix*, Fig. S5).

## Discussion

Despite recent advances in understanding about circadian control of the immune system, relatively little is known of how antibacterial responses are subject to temporal gating. We now show a surprising gain of macrophage function following deletion of BMAL1, resulting in significant reduction in pneumococcal invasion. This protection appeared driven by increased macrophage motility and phagocytic function. The effect was confined to the macrophage and not seen in neutrophils. Further analysis identified changes in RhoA, and cofilin activity resulting from BMAL1 loss, and a major reorganization of macrophage cytoskeletal actin. We used a RhoA inhibitor to investigate function further and observed that very low concentrations, insufficient to impact on wild-type cells, were sufficient to bring down the phagocytosis rate to that seen in wild-type cells, supporting an important role for RhoA activation in the phagocytic gain-of-function phenotype.

Many immune cell types have intrinsic circadian clock activity, leading to time-of-day variation in response (1). Among the immune responses lying under circadian control are acute inflammation, lethality in response to endotoxic shock, allergic reactions, cytokine release, and macrophage phagocytosis (2, 3, 32–34). Susceptibility of mice to infection with *Listeria monocytogenes*, *S. typhimurium*, and *S. pneumoniae* all show a daily rhythm, suggesting a fundamental role for the circadian clock in bacterial defense (5, 7, 18).

A number of cell types and mechanisms have been proposed as mediating temporal control of inflammation and antibacterial response, including barrier epithelium, and cells of the innate immune response, including neutrophils, Ly6C^hi^ monocytes, and resident tissue macrophages (3, 5, 7). Previous attempts to analyze circadian control of phagocytosis have led to conflicting results. Oliva-Ramírez et al. observed small effect sizes and weak circadian rhythmicity in isolated cells, and all circadian signals were lost in freshly isolated macrophages recovered at different times (20). In contrast, Geiger et al. found only ex vivo differences in phagocytosis, but no effects in vivo (21). Therefore, the role of the clock in phagocytosis remains undefined, and the mechanistic links remain undetermined. As we had previously observed striking temporal regulation of pneumococcal pneumonia, which was not explained by *Bmal1* deletion in the club cell, we returned to this model to determine how myeloid timekeeping impacted macrophage antibacterial function. In our studies of macrophages, we have used two cre driver lines: LysM and CX3CR1. Although the CX3CR1 driver has the major advantage of not targeting neutrophils it has been reported to target gene deletion in other myeloid cell types, including mast cells, dendritic cells, and natural killer (NK) cells (35). However, these cell types do not play a prominent role in acute bacterial infections.

The striking observation that loss of the central clock gene *Bmal1* resulted in a major protective effect against bacteraemia in pneumococcal pneumonia was unexpected, as most circadian disruptions impair fitness. Further analysis suggested the effect was due to enhanced motility and phagocytosis, with cells ingesting more bacteria. The effect was confined to macrophages, and not seen in neutrophils, despite similar molecular mechanisms of action. However, macrophages have a strong endogenous clock, coupled to inflammatory signaling cascades, and in neutrophils, timekeeping is driven in part by the vasculature through chemokine actions, and also in part by the actions of BMAL1 driving a gene expression program which affects cellular senescence, tissue migration, and state of inflammatory activation (9, 13). It is important to note that our data revealing the response to pneumococcal infection are surprising, as previously work has shown that loss of BMAL1 in the myeloid lineage results in increased inflammatory responses to bacterial mimics, such as lipopolysaccharide (3). In addition, previous work has shown an increased risk of death from sepsis in response to listeria bacterial infection, when administered by i.p. injection (7), probably resulting from increased cytokine production driving a septic shock phenotype. The key difference here is the sublethal infectious inoculum, administered to the lung, which permits elaboration of an effective immune response, and under these circumstances the gain-of-function macrophage phagocytosis phenotype offers a significant host advantage. The cytokine measurements at 48 h in our model fit with the reduced bacterial burden and mark a move to resolution of the infection.

The discovery of BMAL1-dependent RhoA activation and cytoskeletal actin remodelling is reminiscent of the RhoA changes seen in epithelial/fibroblast cells following circadian manipulation, with impacts on wound healing kinetics (31). Similar cross-coupling between actin dynamics and circadian machinery was reported by Schibler and colleagues, suggesting a signaling pathway from serum response factor through G/F actin to affect circadian phase, and reciprocal signaling contributing to timing of mitosis (28). In our analysis we discovered a widespread reprogramming of cell motility and cytoskeletal gene expression in response to macrophage deletion of BMAL1. This, coupled with the lack of a chemokine directional migration phenotype, suggests the primary mechanism for the phagocytic phenotype is enhanced motility, following loss of organized, cortical actin in the macrophages.

Taken together, we report a gain in macrophage function following deletion of the core clock protein BMAL1, and reveal a mechanism in which BMAL1 acts as a repressor of the immune system, operating as a “brake,” regulating the magnitude of phagocytosis responses following infection. This was not seen with REVERBα manipulation and could not be replicated by physiological circadian manipulations using ambient light changes, although this environmental manipulation may not have sufficiently impacted the macrophage circadian clock to detect an effect. In the absence of BMAL1 expression, REVERBα is greatly reduced, and the cells lack circadian rhythmicity, but in contrast, in the absence of REVERBα, BMAL1 expression is high, and the cells remain circadian rhythmic. Although we previously showed that the exaggerated cytokine responses seen in airway epithelium lacking BMAL1 are phenocopied by disruption of REVERBα, here we document a BMAL1-dependent and REVERBα-independent pathway regulating macrophage migration and phagocytosis. Thus, these two clock proteins have separate but interlocked functions in the elaboration of an inflammatory response, regulating cytokine release and local tissue neutrophilia (REVERB dependent) and macrophage phagocytosis behavior (BMAL1 dependent). We have previously shown that acute TLR-4–mediated cytokine responses to LPS are mediated by rapid degradation of the repressor REVERBα protein [14]. An implication from our current studies, and yet to be tested, is that following infection, enhanced phagocytosis may also be mediated by a similar rapid degradation of BMAL1 protein in macrophage cells. Here we cannot rule out that loss of both REVERBα and REVERBβ may replicate the BMAL1 loss, but equally the BMAL1 effect may be mediated by a non-REVERB pathway, one that relies on direct action of BMAL1 on motility and cytoskeletal gene expression. Indeed, BMAL1 binding was detected close to key motility effector genes, supporting direct BMAL1 regulation.

The circuit involving cytoskeletal actin reorganization has been linked to the circadian clock before, in fibroblasts, but now we find that this confers BMAL1 control to macrophage motility and phagocytosis. The translational implications are clear, with targeted interventions to replicate the macrophage gain of function being attractive approaches to treat prevalent infectious diseases.

## Materials and Methods

### Animals.

Colonies of *LysM-Bmal1*^*−/−*^, *LysM-Rev-Erbα-DBD*^*m*^, and *CX3CR1-Bmal1*^*−/−*^ mice were maintained in the University of Manchester Biological Services Facility (BSF). Where WT-only mice were used, C57BL/6 were purchased from Envigo. All genetically modified mice were created on a C57BL/6 background and housed in 12:12 light/dark (L:D) cycles with food and water supplied ad libitum. All protocols were approved by the University of Manchester Animal Welfare and Ethical Review Body, and the Animals (Scientific Procedures) Act 1986 UK Home Office guidelines were strictly adhered to. Conditional targeted cre-positive mice were sex and age matched with floxed/floxed littermate controls; females aged 10 to 14 wk were used for in vivo experiments and peritoneal exudate cells (PECs) for ex vivo were harvested from male mice. Genotyping was performed on all experimental animals.

### *S. pneumoniae* Infection.

Mice were infected at ZT12 with *S. pneumoniae* (serotype 2, strain D39) (National Collection of Type Cultures [NCTC] 7466), intranasally with 1 × 10^6^ colony-forming units (CFU) per mouse in 20 μL phosphate buffered saline (PBS) under isoflurane anesthesia. Animals were killed 24 or 48 h later. Blood was collected from the femoral artery (into 1:20 heparin). Two methods of lung tissue preparation were used, lung tissue digest and lung homogenate. Lung tissue digest was prepared by digesting minced tissue in a solution of 0.154 mg/mL collagenase I and II (Liberase TM, Roche 05401127001) and 50 μg/mL DNase I (Roche 11284932001). After 30 min at 37 °C, the reaction was stopped with ethylenediaminetetraacetic acid (EDTA) and the digest passed through a 70-μM filter. Homogenate was prepared in Rhino tubes together with six 3.2-mm ball bearings. Tubes were placed in a Storm Bullet blender for 5 min on setting 8. Whole blood and lung homogenate were plated out on Columbia blood-agar plates in serial dilution overnight at 37 °C and CFUs counted the next day.

### Primary Cell Culture.

Cells were collected from mice by peritoneal lavage. PECS were isolated, resuspended in complete Roswell Park Memorial Institute culture medium (RPMI) and seeded at 3 × 10^5^ cell/cm^2^. Cells were washed in media and then used in experiments. For neutrophils, bone marrow was harvested with neutrophils being extracted using the neutrophil enrichment kit (STEMCELL Technologies). Alveolar macrophages were harvested by instilling 1 mL PBS via the trachea into the lungs and this process was repeated three times.

### Phagocytosis Assays.

Different types of phagocytosis assay were used in these studies, in vivo, ex vivo, fixed time point, real-time live cell microscopy, and plate reader.

### In Vivo Phagocytosis Assay.

Mice were injected i.p. with 10 μg (in 200 μL) attenuated *S. aureus* expressing pHrodo green (SAPG). The PECs were harvested and washed twice in PBS and resuspended in FACS buffer (PBS, 2% [bovine serum albumin] 0.1% sodium azide). Samples with excess blood contamination were excluded from the analysis. Flow cytometry was performed immediately on a BD FACS CANTO flow cytometer. Debris was gated out and geometric mean measured in the fluorescein isothiocyanate (FITC) fluorescence channel.

### Flow Cytometry.

A single-cell suspension was generated from pulmonary digest to enable characterization of the pulmonary infiltrate during pneumococcal infection. A live/dead marker was first applied (LifeTechnologies L10119; 1:1,000) in PBS. Antibodies and blocker were made up in FACS buffer (PBS, 2% BSA, and 0.1% sodium azide): Fc receptors were blocked (1:100; anti-CD16/32, eBioscience 14-0161). Antibodies were incubated for 25 min at 4 °C. We used CD11b-PerCPCy5.5 (1:200; clone M1/70, 45-0112), CD11c-APC (1:400; clone N418, 17-0114), Ly6G-FITC (1:100; clone RB6-8C5, 11-5931), and F4/80-PE-Cy7 (1:200; clone BM8, 25-4801) (all eBioscience). After washing, cells were resuspended in 50 μL 1% PFA for 20 min at room temperature (RT). Cells were washed, resuspended in FACS buffer, and analysis was carried out on a BD FACS CANTO flow cytometer. Neutrophils were identified as f4/80^−^CD11b^+^Ly6G^+^. Alveolar macrophages were identified as F4/80^+^CD11c^+^CD11b^lo^. Interstitial macrophages were identified as F4/80^+^CD11c^−^CD11b^hi^.

### Fixed Time Point Phagocytosis Assay.

PECS were seeded at 5 × 10^5^ cells per well in a 24-well plate. After washing, cells were incubated in fresh complete RPMI with 0.1 mg/mL *SAPG*. Targeted cells and floxed controls were incubated at 37 °C and in a separate plate at 4 °C. After 4 h, phagocytosis was stopped with ice-cold PBS wash and cells were detached with 200 μL 1× trypsin EDTA (Sigma) at 37 °C for 15 min, confirmed by microscopy. Cells were washed twice and resuspended in FACS buffer. Flow cytometry was performed immediately on a BD FACS CANTO flow cytometer and mean fluorescent intensity (MFI) was quantified in the FITC channel.

### Real-Time Live Cell Microscopy.

PECS were seeded at 5 × 10^5^ cells per well in a 24-well plate. Fresh RPMI (Sigma) with 10% FBS (Life Technologies) and 1% penicillin/streptomycin (Sigma) was applied. *S. aureus* pHrodoGreen (SAPG) 0.1 mg/mL was added at time 0. Multiple images were acquired on an Eclipse Ti inverted microscope (Nikon), 20× objective and the Nikon green LED FITC channel. A Retiga R6 camera captured the images and the imaging software used was NIS Elements AR (Nikon). Point visiting was used to allow multiple positions to be imaged within the same time course and cells were maintained at 37 °C and 5% CO_2_. Nd2 image files were extracted to TIFF stacks and processed in ImageJ (NIH). The fluorescent intensity in each position was quantified in the green channel in every visual field at every time point. The average value for each genotype was then plotted against time.

### Ex Vivo *S. pneumoniae* Phagocytosis (Gentamicin Protection Assay).

Peritoneal macrophages were infected with pneumococci opsonized with complement (from whole mouse serum) to enhance adherence at a multiplicity of infection (MOI) of 10, incubated at 4 °C for 1 h (to maximize adherence), and then at 37 °C for 3 h (to allow internalization by macrophages) (36). Cultures were washed 3 times in PBS to remove nonadherent bacteria. The cells were then incubated with benzyl-penicillin and gentamicin to kill remaining extracellular bacteria, as described. The cells were lysed in saponin and live intracellular bacteria quantified by the Miles Misra method (37).

### Plate Reader Phagocytosis Assay.

PECS were seeded into a 96-well plate and incubated with 50 µg SAPG. Fluorescence was measured using a Promega Glow max multi detection reader. For the RhoA inhibition prior to phagocytosis, cells were treated with RhoA inhibitor CT04, (Cytoskeleton Inc.) or a vehicle control 2 h before phagocytosis assay with SAPG.

### Nano-String Gene Array.

Peritoneal macrophages were incubated with heat-treated *S. aureus* (wood strain, without protein A) for 1 h. The Promega Reliaprep cell miniprep system (TM370) was used to lyse cells and extract RNA. RNA quality was assessed via Agilent RNA TapeStation. The concentration of the RNA was quantified using a Qubit and the sample concentration was adjusted to 20 ng/μL in 10 μL. The RNA was then run on a NanoString nCounterTM (NanoString Technologies) in conjunction with a custom panel of phagocytosis genes.

### RNA-Seq.

PECS from *LysM-Bmal1*^*−/−*^ and littermate controls (*n* = 5) were seeded at 5 × 10^5^ cells per well in a 24-well plate. After washing peritoneal macrophages were lysed and RNA was extracted with ReliaPrep RNA cell miniprep (Promega). RNA quality was assessed using 4200 Tapestation (Agilent Technologies) and all RNA integrity number (RIN) values were >9.0. Unmapped paired-end sequences from an Illumina HiSeq4000 sequencer were tested by FastQC (http://www.bioinformatics.babraham.ac.uk/projects/fastqc/). Sequence adapters were removed and reads were quality trimmed using Trimmomatic_0.36 (38). The reads were mapped against the reference mouse genome (mm10/GRCm38) and counts per gene were calculated using annotation from GENCODE M21 (www.gencodegenes.org/) using STAR_2.5.3 (39). Normalization, principal component analysis, and differential expression were all determined with DESeq2_1.24.0 (40).

To calculate enrichment of our set of differentially expressed genes (padj < 0.01) with respect to BMAL1 ChIP-Seq data, we used publicly available macrophage BMAL1 ChIP-Seq data from the Cistrome database [Oishi et al. (30), LPS 0 h], and we extracted genesets whose TSSs overlap with BMAL1 peaks for different genomic distances, i.e., ±2 kb to ±1 Mb in seven bins, from the center of the peak. For each such geneset, corresponding to a given genomic distance, a hypergeometric *P* value was calculated for its intersection with the RNA-Seq DE genes, with total number of genes in the genome (mm10) as background.

### Western Blotting.

Total cell protein was isolated from cells using lysis buffer (50 mM Tris, 150 mM NaCl, 1% Triton X-100, 0.2% SDS, supplemented with protease inhibitors). Equal protein quantities were run on an SDS 4 to 20% TGX miniprotean TGX stain-free protein gel (Bio-Rad) and transferred to low fluorescence PVDF membrane (Bio-Rad) via the Trans-Blot Turbo transfer system (Bio-Rad). Membranes were blocked with Odyssey blocking buffer (Li-Cor) for 1 h at room temperature. Samples were probed with antibody overnight at 4 °C (BMAL1-D2L7G, Cell Signaling Technologies 14020). Immunoreactivity was visualized using the Odyssey CLx near-infrared imaging system and fluorescence quantified in ImageStudioLite (Li-Cor).

### G-Actin/F-Actin Ratio.

Expression of G and F actin was determined using the G-actin/F-actin In Vivo Assay Kit by Cytoskeleton, used according to the manufacturer’s instructions (BK037). The protocol separates filamentous from globular actin by ultracentrifugation before detection by immunoblot.

### RhoA Activation Assay.

RhoA activity was measured using the RhoA Activation Assay Biochem Kit by Cytoskeleton, performed according to the manufacturer’s instructions (BK036).

### Transwell Migration Assay.

The 24-well Millicell hanging cell culture inserts (Millipore, MCEP24H48, 8-µm pore) were seeded with 1 × 10^5^ peritoneal macrophages, index wells were treated with 500 ng/mL CCL2 (R&D Systems), and incubated for 4 h. Cells were fixed in 4% PFA for 15 min at RT. Nonmigrated cells were removed with a cotton swab. Cells were stained with crystal violet (5 mg/mL in 2% ethanol) for 30 min at RT. The inserts were washed twice in PBS and excess stain was removed mechanically from the upper side of the membrane. Migrated cells were solubilized in 2% SDS overnight at room temperature and absorbance was read at 560 nm (Glomax plate reader, Promega).

### Migration Microscopy.

Label-free quantitative phase images were generated via Livecyte’s automated Acquire software and Ptychographic reconstruction algorithm. Cells were imaged with a 20× objective lens every 10 min. During imaging, cells were maintained in an environmental chamber at 37 °C with 5% CO_2_ and 95% humidity. Cell tracking was performed automatically with Livecyte Analyze software. Single-cell migration metrics: cell speed and track length, were exported from Analyze and plotted.

### Data Availability Statement.

RNA-Seq data that support the findings of this study have been deposited in ArrayExpress, https://www.ebi.ac.uk/arrayexpress/, with the accession code E-MTAB-8411 (41).

## Supplementary Material

Supplementary File

Supplementary File

Supplementary File

Supplementary File

Supplementary File

Supplementary File
