# Supplementary material for: The clock gene Bmal1 inhibits macrophage motility, phagocytosis, and impairs defense against pneumonia
Source: Proc Natl Acad Sci U S A. 2020 Jan 3;117(3):1543–51. doi: 10.1073/pnas.1915932117 (PMC6983378; doi:10.1073/pnas.1915932117)
Supplement: Supplementary File [file pnas.1915932117.sapp.pdf]

PNAS
www.pnas.org

Supplementary Information for:

The clock gene *Bmal1* inhibits macrophage motility, phagocytosis, and impairs defence against pneumonia.

Gareth. B. Kitchen[1,2,] Peter S. Cunningham[1], Toryn M. Poolman[3], Mudassar Iqbal[1,] Robert Maidstone[3], Matthew Baxter[3], James Bagnall[1], Nicola Begley[1], Tracy Hussell[1], Laura C. Matthews[4], David H. Dockrell[5], Hannah J. Durrington[1,2], Julie E. Gibbs[1], John F. Blaikley[1,2] Andrew S. Loudon[1], David. W. Ray[3]

Corresponding Author email addresses
David. W. Ray : david.ray@ocdem.ox.ac.uk
Andrew Loudon - andrew.loudon@manchester.ac.uk
John Blaikley - john.blaikley@manchester.ac.uk

This PDF file includes:

Figs. S1 to S5
Table S1
Captions for movies S1 to S2
Captions for databases S1 to S3

Other supplementary materials for this manuscript include the following:

Movies S1 to S2
Dataset S1

www.pnas.org/cgi/doi/10.1073/pnas.1915932117

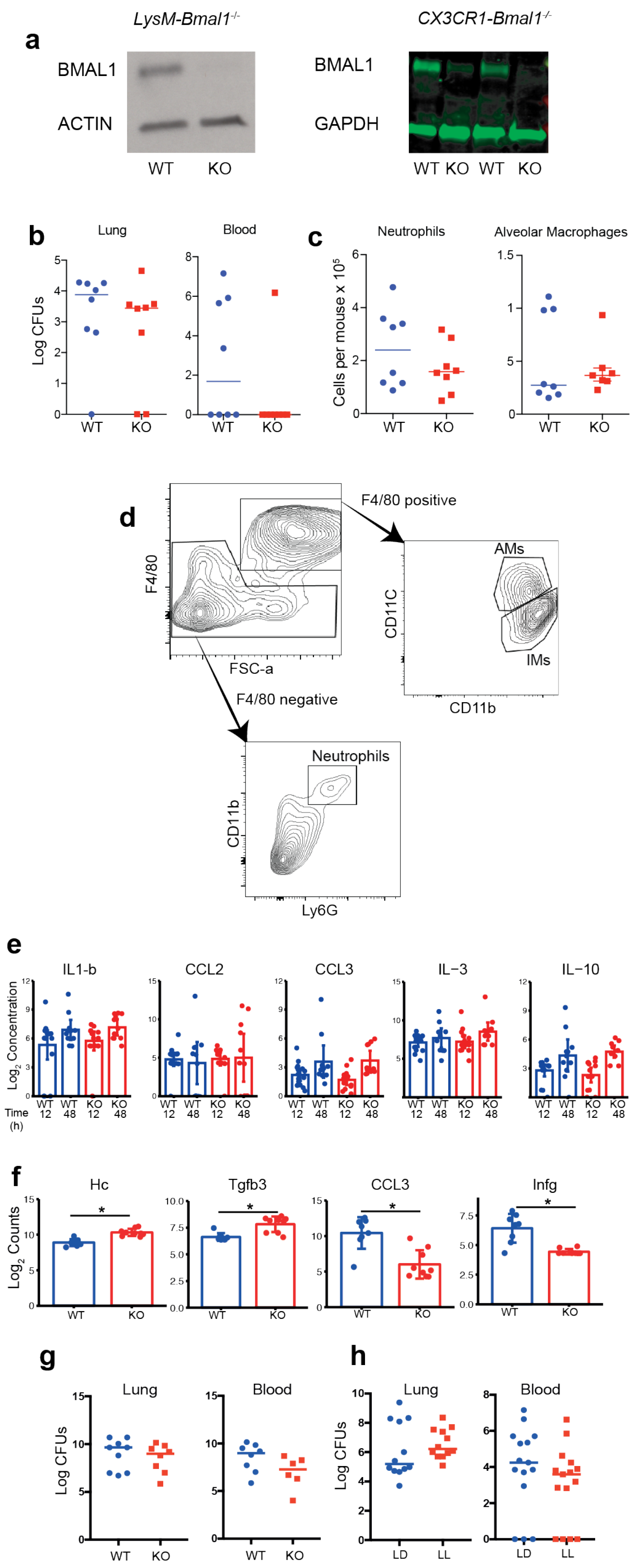

a
LysM-Bmal1-/-
CX3CR1-Bmal1-/-
BMAL1
ACTIN
WT KO
BMAL1
GAPDH
WT KO WT KO
b
Lung
Blood
Log CFUs
WT KO
c
Neutrophils
Alveolar Macrophages
Cells per mouse x 10^5
WT KO
d
F4/80
FSC-a
F4/80 positive
F4/80 negative
AMs
IMs
CD11C
CD11b
Neutrophils
CD11b
Ly6G
e
IL1-b
CCL2
CCL3
IL-3
IL-10
Log2 Concentration
Time (h)
WT 12 WT 48 KO 12 KO 48
f
Hc
Tgfb3
CCL3
Infg
Log2 Counts
WT KO
g
Lung
Blood
Log CFUs
WT KO
h
Lung
Blood
Log CFUs
LD LL

Fig. S1. (a) Left: Immunoblot for BMAL1 from pooled alveolar macrophages from *LysM-Bmal1*$^{-/-}$ and littermate control mice. Right: Immunoblot for BMAL1 from peritoneal exudate cells from *CX3CR1-Bmal1*$^{-/-}$ and littermate control mice (b) *LysM-Bmal1*$^{-/-}$ and floxed, littermate controls were infected with *S. pneumoniae* at ZT12. The bacterial load in the lung and peripheral blood 24 hours later was determined by serial dilution and colony counting. (c) Immune cells were recovered from the lung tissue by digestion (Liberase TM), and analysed by FACS. The populations of neutrophils and alveolar macrophages (AMs) were quantified, median values marked. (d) FACS gating strategy for neutrophils, alveolar macrophages (AMs) and interstitial macrophages (IMs) in Fig. 1. (e) *LysM-Bmal1*$^{-/-}$, and floxed littermate controls were infected with *S. pneumoniae*, and harvested after either 24 or 48 hours. Plasma cytokines from tail bleeds were assessed at 12 and 24 hours following pneumococcal infection quantified by BioPlex. No differences between genotypes (n=7, mean and 95% confidence interval marked). (f) gene changes in whole lung 48 hours following pneumococcal infection. Hc (haemolytic complement). (Differential expression and significance was determined using the limma package * P<0.05 n=8, mean and standard deviation marked) (g) *LysM-Rev-Erbα-DBD*^{m} mice and floxed controls were infected with *S. pneumoniae* the bacterial burden in the lung, and peripheral blood measured at 48 hours. (n= 8-9) Mann-Whitney *U*-test ns p>0.05. (h) WT mice housed in continuous light for 4 weeks (LL), compared to 12:12 L:D (LD) prior to infection. Mice were harvested after 48 hours, and bacterial burden measured. (n=12 per condition) Mann-Whitney *U*-test ns p>0.05.

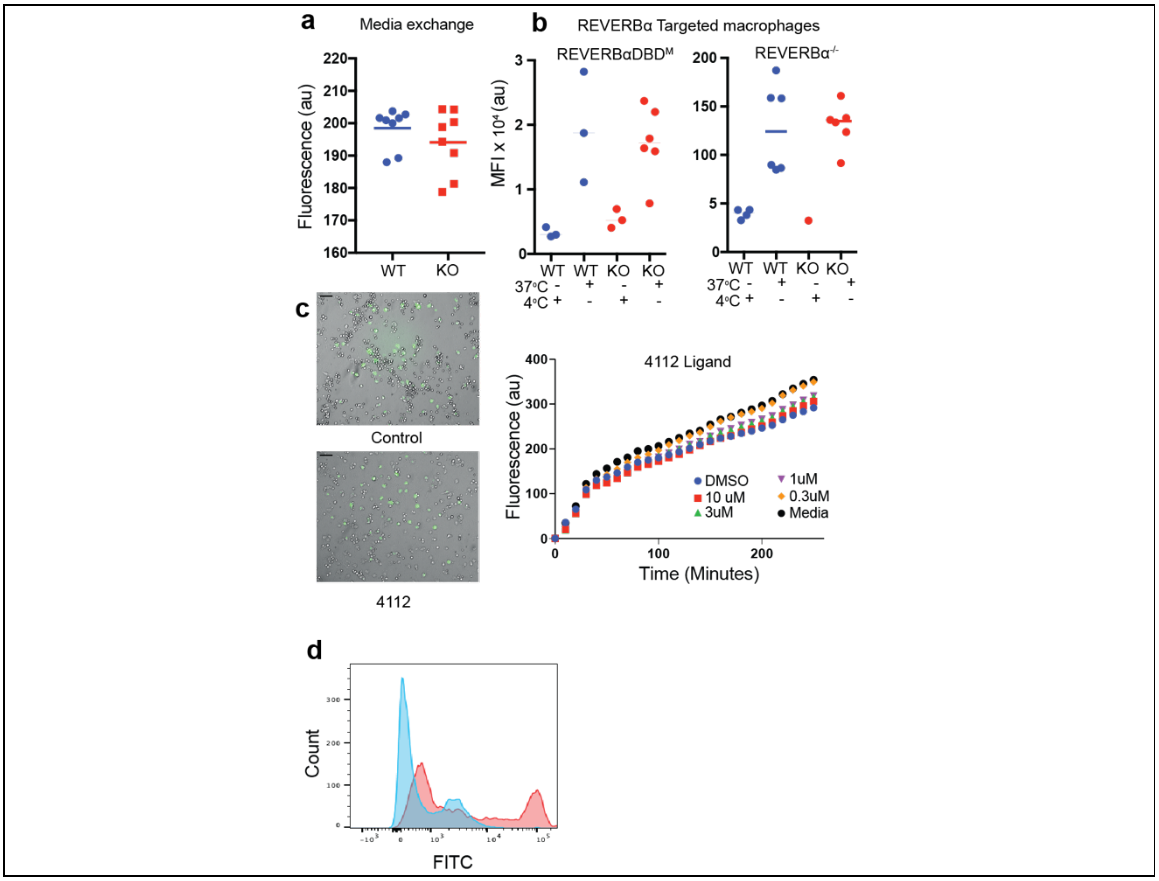

Fig. S2. (a) Conditioned medium recovered from peritoneal macrophage cells of *LysM-Bmal1*$^{-/-}$ mice (KO) and floxed littermate controls. (WT). The media was tested on WT macrophages, and the impact on SAPG phagocytosis measured after 4 hours exposure. No genotype differences were detected (Mann-Whitney *U*-test ns p>0.05).

(b) Peritoneal macrophages recovered from global REVERBα null mice (REVERBa$^{-/-}$), and littermate, wild-type controls; and also from *LysM-Rev-Erbα-DBD*^{m} mice and littermate controls. Purified macrophages were incubated with SAPG for four hours, before FACS analysis of Mean Fluorescent Intensity (MFI). (n=3) (Mann-Whitney *U*-test NS). Cells incubated with *Staphylococcus aureus* pHrodo-green (SAPG) at 4°C to inhibit enzymatic function in the macrophages are also shown.

(c) WT peritoneal macrophages treated with increasing concentrations of REVERBα ligand 4112, DMSO and media controls in the presence of SAPG. (Mean and SEM plotted repeated measures ANOVA NS). Representative microscopy images obtained at the end of the time course are shown.

(d) Representative FACS plot for peritoneal macrophages following treatment with SAPG, showing FITC histogram for control (blue) and treated (red) samples.

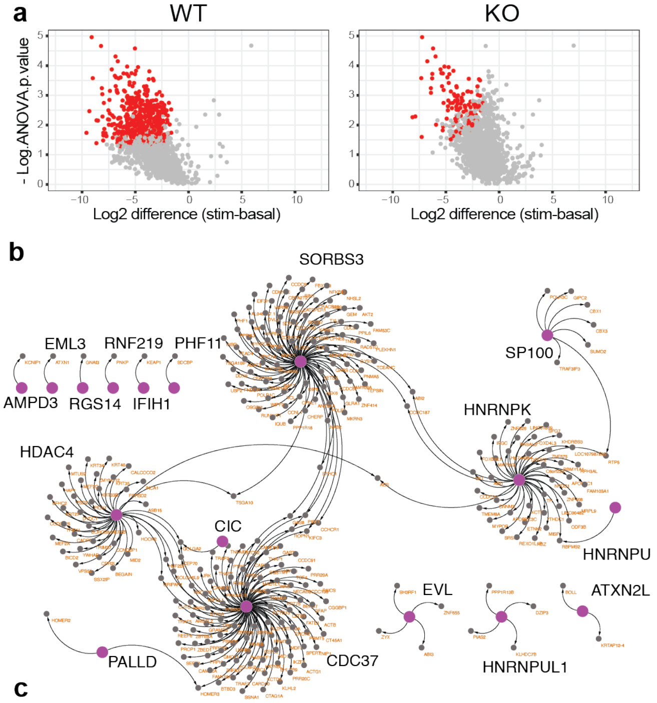

c

Biological Process	P Value
cytoskeleton organization	0.023
**Molecular Function**	
molecular_function	0.0287
binding	1.68E-11
protein binding	1.4E-21
enzyme binding	0.0144
ubiquitin-like protein ligase binding	0.00678
**Cellular Component**	
cytoskeleton	0.0000911
cytoskeletal part	0.0000245
microtubule cytoskeleton	0.000123
microtubule organizing center	0.00529
centrosome	0.0165
cytosol	0.011
**Pathway Enrichment**	
RHO GTPases Activate WASPs and WAVEs	0.029

Fig. S3. Analysis of phosphopeptides regulated by bacterial challenge in peritoneal macrophages.

(a) Peritoneal macrophages from *LysM-Bmal1*$^{-/-}$, and floxed littermate controls for unstimulated culture or post *S. aureus* at 30 minutes. Phosphopeptides were quantified and presented as Volcano plots. The effects of bacterial activation on WT cells, and *Bmal1* null macrophages (KO) are compared. Significant changes in identified phosphorylation sites are shown in red (identified by ANOVA with post hoc test, n=3). Data to the left of the x-axis zero indicates a reduction under stimulated conditions. (b) A protein-protein interaction network was built using the HuRI database based on the phosphoproteins differentially expressed between KO and WT under basal conditions. Differentially abundant phosphoproteins, which have at least one interaction in HuRI, are shown in larger circles (purple) while their interactors are shown in small circles (grey). GO enrichments of this network are shown (c). The full list of potential interacting proteins is in Dataset S1.

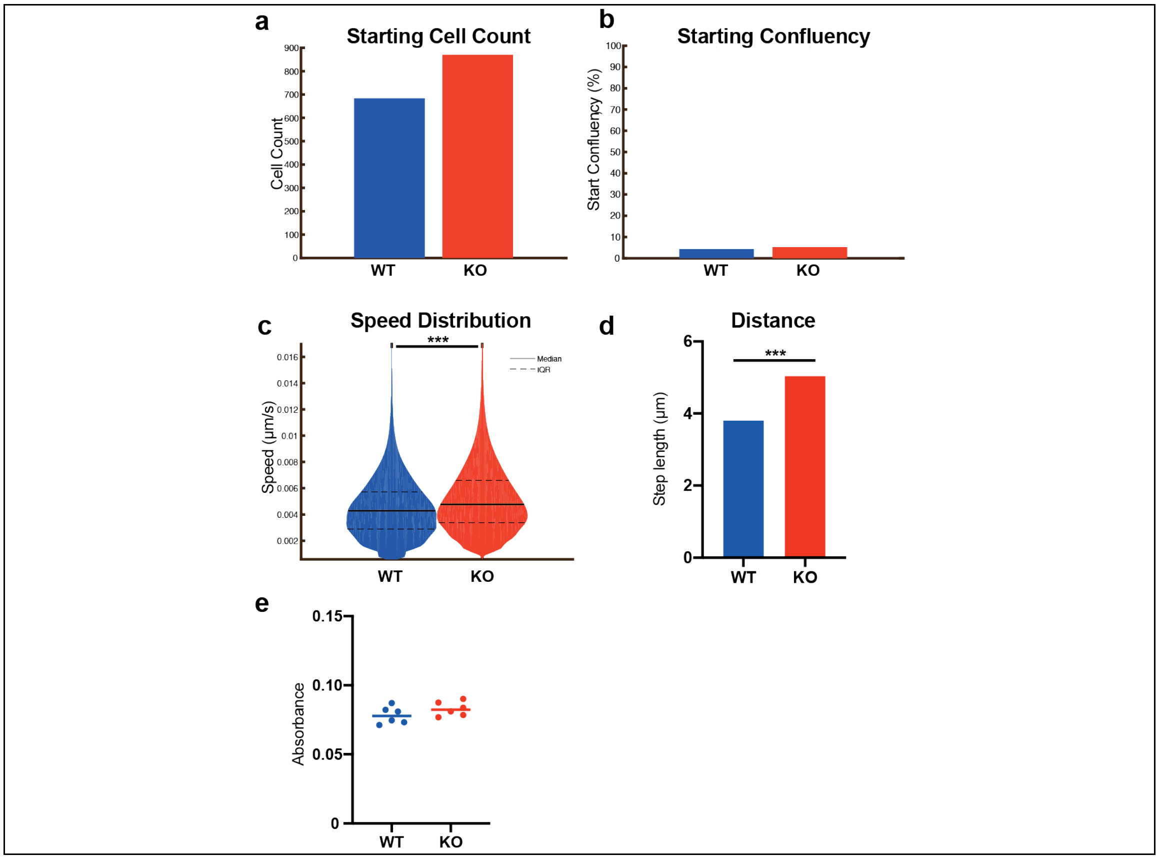

Fig. S4. – Motility of macrophages from *LysM-Bmal1*$^{-/-}$, and littermate controls. Peritoneal macrophages from *LysM-Bmal1*$^{-/-}$, and littermate controls were cultured under control conditions. Macrophages were tracked through spontaneous migration using a Livecyte kinetic cytometer (phasefocus). (a) starting cell count (b) starting confluence (c) Speed distribution unpaired T-test, P value <0.0001 (d) step length unpaired T-test, p value <0.0001. (e) Transwell migration assay overnight serum (Mann-Whitney U-test ns p>0.05).

.

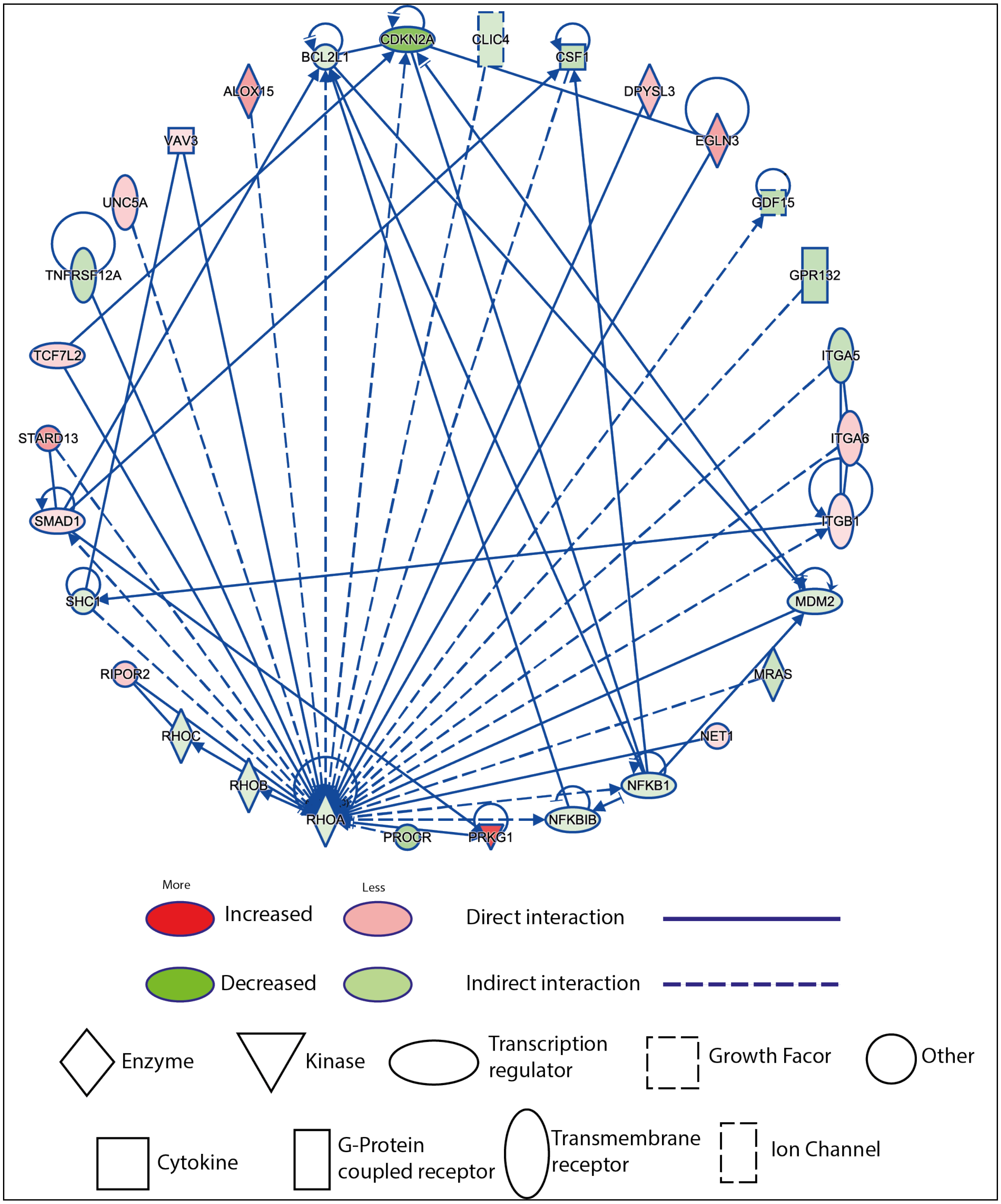

Fig. S5. **RhoA connections to BMAL1 target genes.** Ingenuity Pathway Analysis (IPA) generated diagram showing RhoA connections to differentially regulated cell movement genes. The IPA term "cell movement" was generated from 164 differentially expressed genes in BMAL1 null macrophages (Fig. 3e). In IPA RhoA was added to the gene-set and the direct and indirect connections were generated using the software database of knowledge, resulting in 29 RhoA connected genes shown here.

Table S1. Phagocytosis gene panel used in NanoString gene array, targeting all levels of the phagocytosis pathway

**RECEPTORS**	Ager, Cd14, Cd36, Clec7a (Dectin1), Colec12, Crp, Fas (Tnfrsf6), Fcer1g, Fcgr1, Fcgr2b, Fcgr3, Il1rl1 (St2), Itgam, Itgav, Itgb2, Marco, Mfge8, Myd88, Pecam1, Ticam1 (TRIF), Tlr3, Tlr9.
**RECOGNITION & ENGULFMENT**	Anxa1, C3, Cd44, Cd47, Ceacam3, Crk, Csf1 (Mcsf), Csf2 (GMCSF), Elmo1, Gulp1, Ifng, Mbl2, Mcoln3, Mif, Scarb1, Siglec1, Sirpb1a, Tnf, Wnt5a.
**PHAGOSOME MATURATION**	Cnn2, Iqsec1, Pld1, Rab5a, Rab7, Stx18, Vamp7, Was
**PHAGOSOME PROCESSING**	Calr, Cyp2s1, Nod1 (Card4), Pla2g4a, Pla2g5, Pld2, Serpine1 (PAI-1), Sftpd, Stab2, Tgm2, Tnfsf11 (RANKL).
**SIGNAL TRANSDUCTION**	Adipoq (Acrp30), Axl, Clic4, Csk, Dock1, Dock2, Fyn, Lyn, Mapk14 (p38alpha), Mertk, Msn, Pik3cb, Pip5k1b, Prkce, Pros1, Pten, Rac1, Rac2, Rala, Ralb, Rapgef3, Rhoa, Syk (Sykb), Vav1.

Movie S1. (Separate File)**. WT - Peritoneal macrophage. 3D Reconstruction.** Peritoneal macrophages from floxed littermate controls (WT) cultured under control conditions, live cell imaging with ultra-resolution confocal microscopy, using Airyscan detector and F-actin visualisation with SiR-actin.

Movies S2. (Separate File). **KO Peritoneal macrophage. 3D Reconstruction**. Peritoneal macrophages from *LysM-Bmal1*$^{-/-}$ (KO), were cultured under control conditions, live cell imaging with ultra-resolution confocal microscopy, using Airyscan detector and F-actin visualisation with SiR-actin.

Dataset S1 - The full list of potential interacting proteins from Fig. S3

Dataset S2. List of 164 genes identified in the RNA-SEQ as differentially regulated that lie behind the cell movement term, diseases and functions term in Ingenuity Pathway Analysis.

Dataset S3. 148 genes differentially regulated in the RNA-SEQ and lie within 100KB of BMAL1 peaks.
